# Outcomes of arteriovenous access among cancer patients requiring chronic haemodialysis

**DOI:** 10.1186/s12882-020-01969-5

**Published:** 2020-07-23

**Authors:** Seonjeong Jeong, Hyunwook Kwon, Jai Won Chang, Youngjin Han, Tae-Won Kwon, Yong-Pil Cho

**Affiliations:** 1 Division of Vascular Surgery, Department of Surgery, University of Ulsan College of Medicine and Asan Medical Center, 88 Olympic-ro 43-gil, Songpa-gu, Seoul, 05505 Republic of Korea; 2 Division of Nephrology, Department of Internal Medicine, University of Ulsan College of Medicine and Asan Medical Center, Seoul, Republic of Korea

**Keywords:** Arteriovenous access, Cancer, End-stage kidney disease, Haemodialysis, Outcomes

## Abstract

**Background:**

There are limited data focusing specifically on the types of arteriovenous (AV) access used and outcomes of AV access among cancer patients as a consequence of cancer. We aimed to describe outcomes of AV access among cancer patients requiring chronic haemodialysis, and also to compare outcomes between patients with and without cancer.

**Methods:**

In this single-centre, retrospective, observational cohort study, 84 patients diagnosed with cancer before AV access placement were included; we analysed outcomes of AV access among these patients and compared these outcomes with our previous results. The study endpoints were AV access patency and early failure, defined as AV access abandonment within 12 months after AV access placement.

**Results:**

Various cancer types, stages, and treatments were identified in our analysis. Autologous arteriovenous fistulas (AVFs) were used for 92.9% of this study population. Using our previous results for comparison, we found no significant difference in death-censored primary (*P* = 0.546) and secondary (*P* = 0.266) patency of AV access between patients with and without cancer; however, the rate of early AVF failure was statistically significantly higher among cancer patients (25.6% vs 13.9%; *P* = 0.008), and the most common cause of AVF failure was patient death. The rate of early failure was significantly higher among patients with advanced-stage cancer (59.1%) than among those with early-stage cancer (12.9%) (*P* <  0.001).

**Conclusions:**

Although AV access patency rates were similar among patients with and without cancer in the death-censored analysis, cancer patients were more prone to early AVF failure, mainly due to cancer-associated deaths, and this consideration needs to be carefully balanced against individual patients’ life expectancies, according to cancer type and stage.

## Background

Maintenance dialysis for end-stage kidney disease (ESKD) is part of the typical therapeutic regimen for long-term vital organ replacement, which is considered the most expensive treatment in current medical practice [1]. Despite the growing incidence of cancer worldwide, partly because of the increasing elderly population and improved diagnostic capacity [2–5], dramatic advances in the care of cancer patients have led to significant improvements in outcomes and survival. Therefore, there is an increased need for maintenance dialysis among cancer patients due to the toxicity of cancer therapies and direct kidney damage caused by various solid organ and haematological cancers [6–8]. However, many previous investigations of comorbid cancer patients with ESKD receiving chronic dialysis have focused on estimates of cancer incidence and cancer-related outcomes of the urinary system, including renal cell carcinoma [9–15] and bladder carcinoma [9–12]. There are limited data focusing specifically on the type of arteriovenous (AV) access used and outcomes of AV access among cancer patients as a consequence of cancer, given operative risks, longer maturation times [16–18], and emerging data indicating that early cancer- or ESKD-associated deaths occur frequently [5].

A subset of patients with high-risk cancer who are destined to have limited life spans may be cautioned about undergoing AV access placement. Although maintenance dialysis for cancer patients with ESKD is an important emerging medical issue, there remains considerable heterogeneity in terms of the types of vascular access used. Therefore, this study aimed to describe outcomes associated with AV access for chronic haemodialysis among cancer patients, including the rates and causes of early failure, and also to compare outcomes between patients with and without cancer.

## Methods

### Study design and patient population

In this single-centre, retrospective, observational study, we analysed the data extracted from the medical records of the patients who underwent AV access placement at our hospital from January 2011 through December 2013. Approval for data collection and publication was granted by the institutional review board of the Asan Medical Center (IRB No. 2019–0004), Republic of Korea, which waived the requirement for informed patient consent given the study’s retrospective design.

Between January 2011 and December 2013, a total of 876 patients, ≥20 years old, with ESKD who underwent first upper-extremity AV access placement at our hospital were screened for this study. Of these, 136 patients (15.5%) had comorbid cancer, diagnosed by pathological confirmation at our hospital. To ensure valid assessments and comparisons, we excluded 52 patients (5.9%) diagnosed with cancer after initiating haemodialysis therapy using AV access. A final total of 84 patients (9.6%) were included in the analysis.

The demographic characteristics, risk factors of interest (including diabetes mellitus, hypertension, smoking, and dyslipidaemia), types of AV access, clinical characteristics (including type of cancer, cancer stage, and treatment), and outcomes for all consecutive patients, were recorded in an Excel database (Microsoft Corp., Redmond, WA, USA) and analysed retrospectively. Risk factor variables were defined as previously described [19]. Patients were categorized as having either early- or advanced-stage cancer. Early-stage patients were those with stage 1–2 multiple myeloma according to the International Staging System (ISS) or those with TNM stage 1–2 cancer of other types. Advanced-stage patients were those with stage 3 multiple myeloma or TNM stage 3–4 cancer of other types. For patients with synchronous primary cancers, the more advanced stage was selected as the reference.

### AV access placement

In our study population, a nephrologist was involved in the management of each patient for all medication adjustments, planning of AV access type and haemodialysis initiation, and AV access surveillance [16, 19]. All AV access placement procedures were performed as previously published under local anaesthesia [19–21]. The preferred option for AV access was autologous arteriovenous fistula (AVF) creation, followed by an arteriovenous graft (AVG) placement, at the most distal site of the nondominant arm. Prior to AV access placement, vessel suitability was evaluated by physical examination alone or with supplemental duplex ultrasound according to the necessary criteria, as previously described [19–22]. Types of AV access were categorized as AVF (forearm or upper arm) or AVG (straight or U-shaped forearm graft or straight upper arm graft) [19]. Postoperative surveillance was performed according to the Society for Vascular Surgery’s clinical practice guidelines regarding the surgical placement and maintenance of AV haemodialysis access [16, 19, 23].

### Outcomes of interest and follow-up

AV access patency and early failure were the outcomes of interest. Primary patency was defined as the interval from the time of AV access placement until any intervention designed to preserve or restore blood flow, AV access failure, or study end, whichever occurred first, and secondary patency was defined as the interval from the time of AV access placement until AV access abandonment for any cause, regardless of the number of subsequent interventions [19, 20, 24, 25]. Early failure was defined as AV access abandonment from any cause within 12 months after AV access placement. Maturation failure was defined as AV access inadequate for successful needle cannulation after placement [16, 19, 25, 26]. Previously, we reported patency of AV access (*n* = 641) [19] and AVF (*n* = 524) [16] among chronic haemodialysis patients without cancer from the same registry during the same study period. We conducted a re-analysis to evaluate differences in the death-censored AV access patency rates and the rates and causes of early AVF failure between patients with comorbid cancer and those without cancer.

Last follow-up data were obtained from hospital charts, follow-up physicians, and by means of direct telephone interviews (conducted by S. J.) with the patients or their families to obtain information about each patient’s general health status, the function of the original AV access, and all diagnostic and therapeutic interventions during the interim. In this analysis, only the first event of each outcome was included.

### Statistical analysis

Categorical variables are reported as frequencies or percentages, and continuous variables as means and standard deviations. Categorical variables were compared using the chi-squared test or Fisher’s exact test, as appropriate. Long-term event-free rates were estimated using Kaplan–Meier analysis. *P* <  0.05 was considered statistically significant. Statistical analyses were performed with SPSS Statistics for Windows, version 21.0 (IBM Corp., Armonk, NY, USA).

## Results

### Study sample and baseline characteristics

According to the inclusion and exclusion criteria, 84 cancer patients who underwent first upper-extremity AV access placement at our hospital between January 2011 and December 2013 were consecutively enrolled in this study. The baseline and clinical characteristics of the included patients at the time of cancer diagnosis are presented in Table 1. Their mean age was 62.1 years (median, 63 years; range, 32–87 years), and 72.6% of the patients were men. Relevant comorbidities included hypertension (*n* = 67, 79.8%), chronic kidney disease (CKD) (*n* = 64, 77.1%), and diabetes mellitus (*n* = 35, 41.7%). Prior to the diagnosis of cancer, 64 patients had CKD (77.1%), and of these, 63 (75.0%) were maintained on haemodialysis via central venous catheters (CVCs) before AV access placement. AVF placement (*n* = 78, 92.9%) was performed more often than AVG placement, and there was no mortality or morbidity associated with the AV access placement procedures. The mean time from cancer diagnosis to haemodialysis initiation was 40.3 months (median, 30 months; range, 0–161 months), and to AV access placement was 39.6 months (median, 28 months; range, 0–167 months).

**Table 1:** Baseline demographic and clinical characteristics of the study sample at the time of cancer diagnosis

Variable	Total (*N* = 84)
Age (years)	62.1 ± 11.4
Male	61 (72.6)
Underlying disease	
Hypertension	67 (79.8)
Diabetes mellitus	35 (41.7)
CVD	7 (8.3)
CVA	10 (11.9)
PAOD	2 (2.4)
Chronic kidney disease	64 (77.1)
Smoking	22 (26.2)
On haemodialysis*	63 (75.0)
AVF	78 (92.9)
Cancer to haemodialysis initiation (month)	40.3 ± 41.9 (median, 30; range, 0–161)
Cancer to AV access placement (month)	39.6 ± 42.5 (median, 28; range, 0–167)

Continuous data are expressed as mean ± standard deviation, and categorical data as n (%)

*AV* arteriovenous, *AVF* arteriovenous fistula, *CVA* history of cerebrovascular accident, *CVD* cardiovascular disease, *PAOD* peripheral arterial occlusive disease

^*^Maintenance of haemodialysis via central venous catheter at the time of cancer diagnosis

Various types of cancer were identified in our analysis (Table 2): liver (*n* = 21, 25.0%), kidney (*n* = 19, 22.6%), colorectal (*n* = 11, 13.1%), and others. Of these, synchronous primary cancers were found in five patients: one for each of liver and kidney, liver and colorectal, kidney and breast, lung and stomach, and colorectal and prostate. Cancer stages and treatments are summarized in **Tables S****1****and S****2****.**

**Table 2:** Sites of cancer among the study patients

Cancer site	Total (*N* = 84)
Liver	21 (25.0)
Kidney	19 (22.6)
Colorectal	11 (13.1)
Breast	5 (6.0)
Urinary tract	5 (6.0)
Multiple myeloma	5 (6.0)
Thyroid	5 (6.0)
Lung	4 (4.8)
Prostate	4 (4.8)
Gynaecologic	4 (4.8)
Stomach	2 (2.4)
Others*	4 (4.8)

Data are expressed as n (%)

^*^One for each of gastrointestinal stromal tumour, neuroblastoma, acute myeloid leukaemia, and sarcoma

### Study outcomes and follow-up profiles

The Kaplan–Meier survival analyses of primary and secondary AV access patency, as well as overall survival rates, are presented in **Fig. S****1**. The study outcomes are summarized in Table 3. During the mean follow-up of 39.5 ± 31.5 months (median, 34 months; range, 0–98 months), the uncensored median primary and secondary AV access patency durations were 34.3 months (95% confidence interval [CI], 26.9–41.7 months) and 46.1 months (95% CI, 37.5–54.7 months), respectively; the death-censored median primary and secondary patency durations were 55.8 months (95% CI, 45.8–65.3 months) and 75.9 months (95% CI, 67.1–84.7 months), respectively. Early failure occurred in 21 patients (25.0%), caused by patient death (*n* = 15, 17.9%), maturation failure (*n* = 4, 4.8%), and other factors (*n* = 2, 2.4%). Of the 15 deaths, 12 patients never used their AV access for haemodialysis initiation. All deaths were cancer-related, occurred within 5 months after AV access placement. In terms of cancer site, early failure frequently occurred in association with liver cancer (9/21, 42.9%), kidney cancer (4/19, 21.1%), and multiple myeloma (3/5, 60%) (**Table S****3**). Early failure was relatively more prevalent among multiple myeloma patients. Among the study patients, there were 62 early-stage cancer patients (73.8%) and 22 advanced-stage cancer patients (26.2%). The rate of early failure was significantly higher among patients with advanced-stage cancer (13/22, 59.1% vs 8/62, 12.9%; *P* < 0.001) (Table 4).

**Table 3:** Study outcomes

Outcome	Total (*N* = 84)
Uncensored primary patency	34.3 months (95% CI, 26.9–41.7)
Uncensored secondary patency	46.1 months (95% CI, 37.5–54.7)
Death-censored primary patency	55.8 months (95% CI, 45.8–65.3)
Death-censored secondary patency	75.9 months (95% CI, 67.1–84.7)
Early failure	21* (25.0%)
Patient death	15*
Maturation failure	4
Others	2

*CI* confidence interval

*Includes one patient who received an arteriovenous graft

**Table 4:** Early failure of arteriovenous access according to cancer stage

Cancer stage	No. of patients	Early failure	*P*-value
Early stage*	62 (73.8)	8 (12.9)	< 0.001
Advanced stage†	22 (26.2)	13 (59.1)	
Total	84	21 (25.0)	

Data are expressed as *n* (%)

For patients with synchronous primary cancers, the more advanced stage was selected as the reference

^*^Early stage defined as patients with TNM stage 1–2 or multiple myeloma patients with International Staging System (ISS) stage 1–2

^†^Advanced stage defined as patients with TNM stage 3–4 or multiple myeloma patients with ISS stage 3

### Comparison of death-censored AV access patency and early AVF failure between patients with and without cancer

Among patients without cancer, the death-censored median primary and secondary AV access patency durations were 59.2 months (95% CI, 56.0–62.4 months) and 76.1 months (95% CI, 73.5–78.7 months), respectively. In the death-censored analysis, we observed that the primary (*P* = 0.546) and secondary (*P* = 0.266) AV access patency durations were similar between patients with (*n* = 84) and without (*n* = 641) cancer. Compared with chronic haemodialysis patients without cancer,^16^ the rate of early AVF failure was significantly higher among patients with cancer (20/78, 25.6% vs 73/524, 13.9%; *P* = 0.008). Patient death was the most common cause of early AVF failure among cancer patients (14/78, 17.9% vs 21/524, 4.0%; *P* = 0.001), whereas maturation failure was the most common cause among patients without cancer (4/78, 5.1% vs 46/524, 8.8%; *P* = 0.001) (Table 5).

**Table 5:** Early failure of arteriovenous fistulas among patients with cancer and those without cancer

	Cancer patients (*N* = 78)	Patients without cancer (*N* = 524)	*P*-value
Early failure	20 (25.6)	73 (13.9)	0.008
Patient death	14 (17.9)	21 (4.0)	0.001
Maturation failure	4 (5.1)	46 (8.8)	0.001
Others	2 (2.6)	6 (1.1)	0.680

Data are expressed as *n* (%)

## Discussion

In Korea, the annual growth rate of the number of dialysis patients has been as high as 9% in recent years, owing largely to the expansion of the diabetic and elderly populations, and about half of dialysis patients have been undergoing dialysis for more than 10 years [1]. Despite the lack of consensus, there is general agreement that CKD patients have a greater risk of cancer than the general population [1–5, 27]. Furthermore, in addition to the occurrence of acute kidney injury (AKI) following cancer treatment with surgery, radiotherapy, or chemotherapy, the increasing incidence of CKD and increased life spans of cancer patients can lead to the progression of CKD and need for AV access placement for lifelong haemodialysis [6, 28]. Although there are racial and ethnic differences in environmental and genetic factors, comorbidities, and the incidence and types of cancer, the changing patient demographics and increasing proportion of elderly patients may further increase both ESKD and active cancer. However, because of the considerable heterogeneity in cancer types and their prognoses, and various management strategies according to cancer type, there currently are no standard recommendations regarding the types of vascular access to use for haemodialysis in cancer patients, including CVCs.

In this study, we retrospectively analysed limited data from a single centre; the study sample consisted only of patients with comorbid cancer before AV access placement, regardless of whether haemodialysis was initiated via CVC, to focus on accurate outcomes of chronic haemodialysis AV access among cancer patients, and to compare these outcomes with those of patients without cancer. In our analysis, various cancer types, stages, and treatments were identified, and autologous AVFs were used for 92.9% of the patients in this sample. Compared with our previous results from the same registry during the same study period [16, 19], there was no significant difference in death-censored primary and secondary AV access patency duration between patients with and without cancer. However, the rate of early AVF failure was significantly higher among patients with comorbid cancer, and the most common cause of failure was cancer-associated patient death.

Of the various cancer types, haematological malignancies have been associated with the greatest risk of AKI development in most case series, and multiple myeloma patients are particularly prone to developing AKI [6]. About 50% of newly diagnosed multiple myeloma patients have some degree of renal impairment at presentation, up to 20% have severe AKI, and about 1–5% may require dialysis, whereas severe renal impairment is associated with a high risk of early death and other complications [6, 29, 30]. In our analysis, early failure occurred more frequently among multiple myeloma patients; all three cases of early failure among multiple myeloma patients were due to cancer-associated deaths, which occurred at 3, 5, and 7 months after AVF placement. The early failure rate among advanced-stage cancer patients was significantly higher than that among early-stage cancer patients. For cancer patients with favourable prognoses, well-functioning AV access could be among the most important factors influencing quality of life and longevity; however, for patients who have limited life spans, the type of AV access, including CVC, is less likely to have any association with clinically significant events.

Dramatic advances in the care of cancer patients have led to significant improvements in outcomes and survival. However, a consequence of these advanced therapies has been increased AKI incidence in many forms secondary to the underlying cancer or the effects of anti-neoplastic therapies. The causes of AKI among cancer patients have become more complex and multi-factorial, with an increased risk of AKI associated with advanced cancer stage, haematological malignancies, older age, the presence of underlying CKD, and diabetes mellitus [6]. Two recent studies reported overall 1-year incidence rates of AKI among cancer patients between 11 and 20%, with higher levels of risk among patients with kidney cancer, liver cancer, and multiple myeloma [7, 8]. Although uncertainty remains about whether the relationship between AKI and the risk of ESKD is causal or whether it is confounded by the presence of other chronic conditions [28], many observational studies have consistently described this relationship [31, 32]. Several studies have demonstrated that some, but not all patients who develop AKI go on to develop new or progressive CKD and ESKD [31, 32]. In a large Danish cohort of patients with both cancer and AKI, 5% of patients required maintenance dialysis within 1 year of AKI onset, and this was associated with decreased survival [7, 8]. Among patients with cancer, increased mortality might be due to the toxicity of cancer therapies or direct kidney damage caused by various solid organ and haematological cancers [6].

Our study had some limitations. First, owing to its retrospective and observational design, using single-centre registry data from a relatively small number of patients with a short follow-up duration, this study was potentially affected by selection and information biases. A substantial number of cancer patients with limited life spans receiving haemodialysis via CVC were excluded from this study. Furthermore, the decisions regarding the type of AV access were mainly made by physicians, based on the expected rate of decline in renal function, vessel diameter, and vessel quality. Second, we arbitrarily categorized cancer patients as having either early- or advanced-stage cancer, according to their cancer stages, because of the considerable heterogeneity in cancer types and their prognoses. Third, our study cohort comprised only subjects of Asian descent with various cancer types; thus, because there may be racial or ethnic differences in terms of incidence and types of cancer, caution should be applied when interpreting and generalizing these results. Fourth, based on the small sample size of this single-centre cohort, this study was likely underpowered with limitations to the overall generalizability of our results. Finally, as with all observational studies, our study does not confirm a causal relationship between cancer and outcomes of AV access. Additional large cohort studies are required.

## Conclusions

Despite similar death-censored AV access patency rates between patients with and without cancer, this study has highlighted that cancer patients undergoing AVF placement for chronic haemodialysis are susceptible to potential early failure, mainly due to cancer-associated deaths, and this needs to be carefully balanced against individual patients’ life expectancies, according to cancer type and stage. Considering that there is a paucity of consistent data regarding the outcomes of vascular access placement for chronic haemodialysis among cancer patients, our findings may inform careful and individualized decision-making in this clinical context.

## Supplementary information

**Additional file 1: Table S1.** Cancer stages among the study patients. This table contains categorization of the study patients as having either early- or advanced-stage cancer. **Table S2.** Cancer treatments among the study patients. This table contains various cancer treatment modalities according to cancer sites. **Table S3.** Early failure according to cancer sites. This table contains the detailed data of early failure according to cancer sites.

**Additional file 2: Figure S1.** Kaplan–Meier analyses of (A) primary and (B) secondary patency of arteriovenous access, and (C) overall survival rates of the study sample.

## Data Availability

The datasets used and/or analyzed during the current study are available from the corresponding author upon reasonable request.

## Abbreviations

AKI: Acute kidney injury
AV: Arteriovenous
AVF: Autologous arteriovenous fistula
AVG: Arteriovenous graft
CVC: Central venous catheter
CKD: Chronic kidney disease
ESKD: End-stage kidney disease
