# Supplementary material for: Outcomes of arteriovenous access among cancer patients requiring chronic haemodialysis
Source: BMC Nephrol. 2020 Jul 23;21:297. doi: 10.1186/s12882-020-01969-5 (PMC7379794; doi:10.1186/s12882-020-01969-5)
Supplement: Supplementary file 1 — Additional file 1: Table S1. Cancer stages among the study patients. This table contains categorization of the study patients as having either early- or advanced-stage cancer. Table S2. Cancer treatments among the study patients. This table contains various cancer treatment modalities according to cancer sites. Table S3. Early failure according to cancer sites. This table contains the detailed data of early failure according to cancer sites. [file 12882_2020_1969_MOESM1_ESM.docx]

**Table S1** Cancer stages among the study patients

| Cancer site |  | Early cancer  TNM stage 1–2 | Advanced cancer  TNM stage 3–4 |
| --- | --- | --- | --- |
| Liver* | 21 | 16 | 5 |
| Kidney† | 18 | 15 | 3 |
| Colorectal† | 10 | 9 | 1 |
| Breast | 4 | 2 | 2 |
| Urinary tract | 5 | 5 | 0 |
| Multiple myeloma‡ | 5 | 1 | 4 |
| Thyroid | 5 | 5 | 0 |
| Lung† | 4 | 3 | 1 |
| Prostate | 3 | 3 |  |
| Gynaecologic | 4 | 2 | 2 |
| Stomach | 1 | 1 |  |
| Others§ | 4 |  | 4 |
| Total | 84 | 62 (73.8) | 22 (26.2) |

Data are expressed as n (%).

For patients with synchronous primary cancers, the more advanced stage was selected as the reference: included *two synchronous primary cancers and †one synchronous primary cancers

‡Cancer stage according to International Staging System (ISS): early cancer defined as ISS stage 1–2 and advanced cancer defined as ISS stage 3

§One for each of gastrointestinal stromal tumour, neuroblastoma, acute myeloid leukaemia, and sarcoma

**Table S2** Cancer treatments among the study patients

| Cancer site | Total (N = 84) | Treatment |  |
| --- | --- | --- | --- |
| Liver* | 21 | Surgery | 10 |
|  |  | Surgery / Chemotherapy | 3 |
|  |  | Chemotherapy | 7 |
|  |  | Not available‡ | 1 |
| Kidney† | 18 | Surgery | 15 |
|  |  | Surgery / Chemotherapy | 3 |
| Colorectal† | 10 | Surgery | 7 |
|  |  | Surgery / Chemotherapy | 3 |
| Breast | 4 | Surgery / Chemotherapy / Radiotherapy | 5 |
| Urinary tract | 5 | Surgery | 4 |
|  |  | Surgery / Chemotherapy / Radiotherapy | 1 |
| Multiple myeloma | 5 | Chemotherapy | 4 |
|  |  | Not available‡ | 1 |
| Thyroid | 5 | Surgery | 4 |
|  |  | Surgery / Radiotherapy | 1 |
| Lung† | 4 | Surgery | 2 |
|  |  | Chemotherapy / Radiotherapy | 2 |
| Prostate | 3 | Surgery | 3 |
| Gynaecologic | 4 | Surgery / Chemotherapy | 4 |
| Stomach | 1 | Surgery | 1 |
| Others† | 4 | Surgery or Chemotherapy or Radiotherapy | 4 |

Data are expressed as n.

For patients with synchronous primary cancers, the more advanced stage was selected as the reference: included *two synchronous primary cancers and †one synchronous primary cancer

‡Cancer treatment at other hospitals

§One for each of gastrointestinal stromal tumour, neuroblastoma, acute myeloid leukaemia, and sarcoma

**Table S3** Early failure according to cancer sites

| Cancer site | Total (N = 21) |
| --- | --- |
| Liver* | 9† (42.9) |
| Kidney‡ | 4 (21.1) |
| Colonrectal‡ | 1 (9.1) |
| Breast* | 1 (20.0) |
| Urinary tract | 0 (0) |
| Multiple myeloma | 3 (60.0) |
| Thyroid | 0 (0) |
| Lung | 1 (25) |
| Prostate | 0 (0) |
| Gynaecologic | 1 (25) |
| Stomach | 1 (50.0) |
| Others§ | 2 (50.0) |

Data are expressed as n (%).

Synchronous primary cancers were found in two patients: one for each of liver and kidney, and kidney and breast.

*Includes one arteriovenous graft

†Includes one early failure in a patient with an arteriovenous graft

‡Includes two arteriovenous grafts

§One for each of gastrointestinal stromal tumour, neuroblastoma, acute myeloid leukaemia, and sarcoma
