# Supplementary material for: Outcomes of arteriovenous access among cancer patients requiring chronic haemodialysis
Source: BMC Nephrol. 2020 Jul 23;21:297. doi: 10.1186/s12882-020-01969-5 (PMC7379794; doi:10.1186/s12882-020-01969-5)
Supplement: Supplementary file 2 — Additional file 2: Figure S1. Kaplan–Meier analyses of (A) primary and (B) secondary patency of arteriovenous access, and (C) overall survival rates of the study sample. [file 12882_2020_1969_MOESM2_ESM.pdf]

Figure S1 Kaplan–Meier analyses of (A) primary and (B) secondary patency of arteriovenous access, and (C) overall survival rates of the study sample

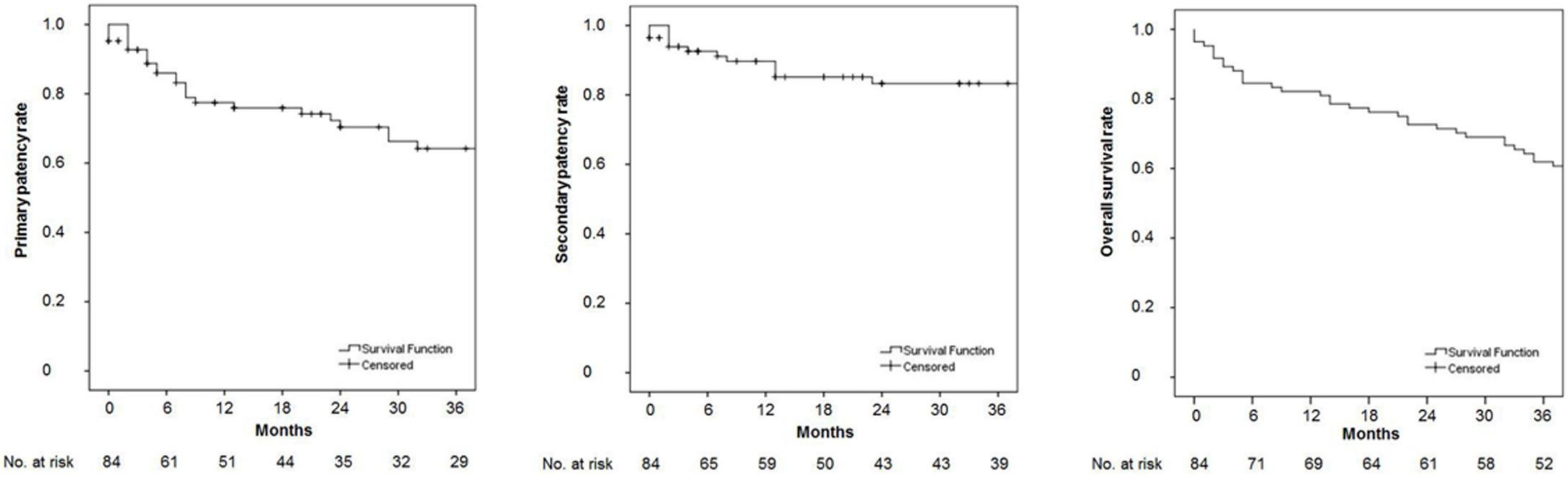

(A) **(B)** **(C)**
